# Bilingualism and Musicianship Enhance Cognitive Control

**DOI:** 10.1155/2016/4058620

**Published:** 2015-12-27

**Authors:** Scott R. Schroeder, Viorica Marian, Anthony Shook, James Bartolotti

**Affiliations:** Department of Communication Sciences and Disorders, Northwestern University, 2240 Campus Drive, Evanston, IL 60208, USA

## Abstract

Learning how to speak a second language (i.e., becoming a bilingual) and learning how to play a musical instrument (i.e., becoming a musician) are both thought to increase executive control through experience-dependent plasticity. However, evidence supporting this effect is mixed for bilingualism and limited for musicianship. In addition, the combined effects of bilingualism and musicianship on executive control are unknown. To determine whether bilingualism, musicianship, and combined bilingualism and musicianship improve executive control, we tested 219 young adults belonging to one of four groups (bilinguals, musicians, bilingual musicians, and controls) on a nonlinguistic, nonmusical, visual-spatial Simon task that measured the ability to ignore an irrelevant and misinformative cue. Results revealed that bilinguals, musicians, and bilingual musicians showed an enhanced ability to ignore a distracting cue relative to controls, with similar levels of superior performance among bilinguals, musicians, and bilingual musicians. These results indicate that bilingualism and musicianship improve executive control and have implications for educational and rehabilitation programs that use music and foreign language instruction to boost cognitive performance.

## 1. Introduction

By examining the effects of various experiences on cognitive performance, we can gain a better understanding of the plasticity of the mind and brain. This understanding can, in turn, be used to develop high-quality educational and rehabilitation programs. Here, we consider two common experiences that may improve certain aspects of cognition: learning how to speak a second language (becoming a bilingual) and learning how to play a musical instrument (becoming a musician). In previous work, bilinguals and musicians were found to have increased domain-general executive control, as evidenced by superior performance on nonlinguistic, nonmusical, visual-spatial tasks that involved attending to a relevant and informative feature of a stimulus while ignoring an irrelevant and misinformative feature [1, 2]. However, the evidence for increased executive control is mixed in bilinguals [3–5] and limited in musicians [6, 7], suggesting a need for more research to confirm or deny these cognitive benefits. It is also unknown how the combination of bilingualism and musicianship affects executive control. In the current study, we investigate these issues by testing bilinguals, musicians, bilingual musicians, and controls on a Simon task that assesses interference suppression (the ability to ignore an irrelevant and misinformative cue).

Bilinguals gain experience using interference suppression during language processing because of their need to prevent the nontarget language from interfering while using the target language. For example, during speech comprehension, both English-Spanish bilinguals and English monolinguals mentally activate similar-sounding words in the target language, such as the English word carton, when hearing the English word “carpet” [8, 9]. Bilinguals, however, also activate similar-sounding words in their other language, such as the Spanish word cartera (i.e., wallet), when hearing the English word “carpet” [9–11]. This parallel activation is instantiated in bilingual models of both spoken word comprehension (e.g., BLINCS [12]) and written word comprehension (e.g., BIA+ [13]). Similarly, during speech production, both languages become activated in parallel [14], consistent with Green's inhibitory control model [15]. Due to activation of both languages during comprehension and production, bilinguals accrue extensive practice inhibiting interference from the nontarget language. Through experience-dependent plasticity, this practice may lead to better domain-general interference suppression [1].

As evidence, a recent study tested bilingual and monolingual younger adults on a numerical Stroop task, in which participants viewed items on a screen (e.g., the number 11) and had to indicate how many distinct items were presented (in this case, 2, because each digit is a different item) [16]. In some trials, called incongruent trials, the quantity expressed by the items was incongruent with the number of items (e.g., 33). Interference suppression was required on these trials in order to ignore the irrelevant feature (i.e., the quantity expressed by the items), which conflicted with the correct response. In other trials, called neutral trials, letters appeared on the screen (e.g., GG) and interference suppression was not required, because the presented items did not express a quantity that was at odds with the number of items. In still other trials, referred to as congruent trials, the expressed quantity and number of items matched (e.g., 333) and therefore interference suppression was not required on these trials either. To measure the ability to suppress interference on the incongruent trials, an interference effect was calculated by subtracting response times on neutral trials (a baseline condition) from response times on incongruent trials. The interference effect was smaller for bilingual younger adults relative to monolingual younger adults, indicative of better interference suppression in bilinguals. A bilingual advantage in interference suppression has been observed in other studies as well [17, 18]. Furthermore, better interference suppression in bilinguals compared to monolinguals has been linked to neural differences in regions associated with executive control [19].

However, some studies have failed to find better interference suppression in bilinguals, particularly in young adults, but also in children and older adults [4, 5, 20–22]. One possible reason why some studies may fail to find a bilingual effect is the use of inadequate measures of interference suppression. Many commonly used executive control tasks include incongruent trials and congruent trials but do not include neutral trials (i.e., baseline control trials). When neutral trials are included, interference suppression can be accurately calculated by subtracting response times on neutral trials (i.e., baseline control trials) from response times on incongruent trials (yielding an interference effect). Otherwise, interference suppression is calculated by subtracting response times on* congruent trials* from response times on incongruent trials (yielding a Simon effect). The Simon effect calculation does not provide a pure measure of interference suppression because it is influenced by how much participants benefit from the helpful congruent cue (i.e., congruent facilitation). Bilinguals sometimes benefit slightly more from a congruent cue than monolinguals [16, 23], which has the numerical effect of increasing the Simon effect score (indicative of poorer performance). The Simon effect measure may therefore mask a true bilingual advantage in interference suppression, and so it is critical that assessments of cognitive advantages include a neutral baseline condition.

Similar to bilingualism, musicianship may also enhance interference suppression through experience-dependent plasticity, though the evidence is significantly more limited, and the source of this potential enhancement is less clear. One possibility is that musical experience enhances interference suppression partly through the same type of mechanism as bilingualism. Recently, theories of music comprehension have drawn parallels to language comprehension and have posited that, as a melody unfolds, other melodies that are consistent with initial notes of the target melody become activated (similar to activation of similar-sounding words in language processing) [24, 25]. Music comprehension would therefore involve a need to ignore misinformation (i.e., activated but incorrect melodies), a notion that is supported by studies indicating activation of frontal executive areas during music listening [26, 27]. This practice inhibiting interference from nontarget melodies during music comprehension may lead to enhancements in interference suppression.

Evidence for enhanced interference suppression in experienced musicians comes from a recent study, in which professional musicians were found to have smaller Stroop effects than amateur musicians [28]. Additionally, in a study with older adults, smaller Stroop effects and Simon effects were observed in musicians relative to nonmusicians ([29]; see also [6] for a similar finding in younger adults). Furthermore, musicians do not show the same age-related declines in executive control areas (e.g., dorsolateral prefrontal cortex and inferior frontal gyrus) as nonmusicians do [30]. These results suggest that musicians may have better interference suppression than nonmusicians, but more research is needed to confirm this finding, given the small number of studies. In the current study, we provide additional data on musicians' executive control abilities.

We also consider the executive control abilities of bilingual musicians. To our knowledge, no previous studies have considered the combined effect of bilingualism and musicianship on interference suppression. If both the bilingual and musician advantages do exist, they may be additive, resulting in even stronger benefits in bilingual musicians. However, bilingualism and musicianship were found to combine nonadditively in a study assessing task-switching abilities [7]. This finding suggests an alternative hypothesis (that the enhancements in interference suppression may not be larger in bilingual musicians). Advantages in interference suppression due to bilingualism or musicianship alone could already place younger adults at their cognitive peak, precluding any extra gains from acquiring both experiences. In line with this reasoning, bilinguals who have other executive control enhancing traits (e.g., video game experience or high socioeconomic status) do not show further gains over bilinguals who do not have these experiences [31, 32], suggesting that bilinguals may reach a ceiling level that is resistant to further plasticity. We evaluate these alternative possibilities in the current study.

In sum, previous research suggests that, through experience-dependent plasticity, bilinguals and musicians may develop enhanced interference suppression, but the evidence is mixed for bilinguals and limited for musicians. Moreover, it remains unknown how the combination of bilingualism and musicianship affects interference suppression. In the current study, we examined interference suppression in bilinguals, musicians, and bilingual musicians. We tested a large sample of participants with varying linguistic and musical backgrounds. Based on bilingual and musical proficiency, four groups were formed: bilinguals, musicians, bilingual musicians, and controls (nonbilinguals and nonmusicians). Each group performed the Simon task, which is a nonlinguistic, nonmusical, visual-spatial task that can be used to assess domain-general executive control abilities in bilinguals and musicians [1, 29]. The Simon task involves responding to the color of a rectangular box (pressing a key on the right if blue and a key on the left if brown), while the irrelevant location of the box is misinformative (incongruent trials), informative (congruent trials), or uninformative (neutral trials). The difference in response time between incongruent and neutral trials, called the interference effect, was used to assess interference suppression. We also considered the facilitation effect (difference between neutral and congruent trials) and Simon effect (difference between incongruent and congruent trials), both of which may be influenced by bilingual and/or musical experience [16, 28].

## 2. Method

### 2.1. Participants

**Figure 1:**
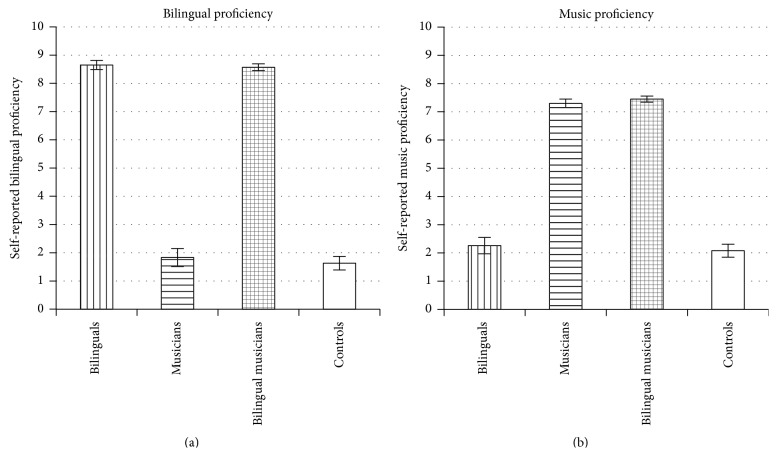
Mean bilingual (a) and music (b) proficiencies for bilinguals, musicians, bilingual musicians, and controls. Bilingual proficiency (a) represents a self-reported measure of ability in the participant's second-most proficient language, while music proficiency (b) represents a self-reported measure of ability in the participant's first-most proficient instrument. Error bars represent the standard error of the mean.

Participants were 219 young adults (mean age = 21.9) with a range of linguistic and musical backgrounds. These participants were recruited through classes and flyers at a university in the United States and were then tested in a university research lab. After data collection, the participants were divided into four groups:* bilinguals* (high bilingual proficiency, low music proficiency; *N* = 43),* musicians* (low bilingual proficiency, high music proficiency; *N* = 42),* bilingual musicians* (high bilingual proficiency, high music proficiency; *N* = 69), and* controls* (low bilingual proficiency, low music proficiency; *N* = 65). Groups were formed using median splits based on bilingual proficiency (defined as self-rated proficiency in understanding their second best language on a 0–10 scale) and music proficiency (defined as self-rated proficiency in playing their best instrument on a 0–10 scale). Ratings were obtained using the* Language Experience and Proficiency Questionnaire* (*LEAP-Q*; [33]) and a music questionnaire ([34], originally adapted from [35]). The question used to measure language proficiency was “*On a scale from zero to ten, please select your level of proficiency in understanding this language*” and the question used to measure music proficiency was “*What is your skill level in playing this instrument/singing?*” with the scale in both cases being “*0-none, 1-very low, 2-low, 3-fair, 4 slightly less than mediocre, 5-mediocre, 6-slightly more than mediocre, 7-good, 8-very good, 9-excellent, 10-perfect.*” (Proficiency in* understanding* the second language was used to represent bilingual proficiency because previous research suggests that receptive abilities (potentially more so than expressive abilities) play a critical role in the development of the bilingual advantage in executive control (e.g., [8]). For example, preverbal bilingual infants show advantages despite a lack of expressive skills (e.g., [36]). In addition, significant correlations between receptive language tasks and executive control performance have been found in younger bilingual adults (e.g., [37]). It should also be noted that, in the current sample, the correlation between first and second language receptive and expressive proficiency was very high (*r* = 0.95).) Mean bilingual and music proficiencies for each of the 4 groups are displayed in Figure 1. Mean bilingual proficiency for each group was as follows: bilinguals = 8.65 (SE = 0.16), musicians = 1.83 (SE = 0.32), bilingual musicians = 8.57 (SE = 0.12), and controls = 1.63 (SE = 0.24). Mean music proficiency for each group was as follows: bilinguals = 2.26 (SE = 0.29), musicians = 7.30 (SE = 0.15), bilingual musicians = 7.45 (SE = 0.11), and controls = 2.08 (SE = 0.23). A one-way ANOVA with bilingual proficiency as the dependent measure revealed an effect of Group, *F*(3,215) = 350.10, *p* < 0.05, with bilinguals and bilingual musicians reporting significantly higher bilingual proficiencies than musicians and controls (*p*s < 0.05, Bonferroni corrected). A one-way ANOVA with music proficiency as the dependent measure also revealed an effect of Group, *F*(3,215) = 234.77, *p* < 0.05, with musicians and bilingual musicians reporting significantly higher music proficiencies than bilinguals and controls (*p*s < 0.05, Bonferroni corrected). Similarly, for current percentage of time using a second language, bilinguals (mean = 24.8%) and bilingual musicians (mean = 23.7%) reported more current usage than musicians (mean = 2.1%) and controls (mean = 4.5%), *F*(3,215) = 45.66, *p* < 0.05, pairwise comparison *p*s < 0.05, Bonferroni corrected. Likewise, for current hours per week of playing an instrument, musicians (mean = 3.1) and bilingual musicians (mean = 2.7) reported more current usage than bilinguals (mean = 0.1) and controls (mean = 0.4), *F*(3,215) = 8.95, *p* < 0.05, pairwise comparison *p*s < 0.05, Bonferroni corrected.

The approach of using median splits of proficiency to categorize participants into groups for the analyses was chosen over an approach of using continuous proficiency scores, for two reasons. The first is that both bilingual proficiency and music proficiency were bimodally distributed (into a high and low group), as determined by Hartigan's Dip Statistic (bilingual proficiency, HDS = 0.11, *p* < 0.05; music proficiency, HDS = 0.07, *p* < 0.05). The second is that, by dividing participants into groups, our findings can be more easily connected to other research in the field, as most research in this field uses a categorical group approach (e.g., [1, 16, 23]). Nevertheless, we supplement this categorical approach with a continuous approach by conducting regression analyses of bilingual proficiency and music proficiency scores (see Section 2.3).

Bilinguals, musicians, and bilingual musicians had experience with a large variety of languages and/or instruments. This diverse group of participants represents the wide range of linguistic and musical experiences that exists in the real world, thereby increasing the external validity of the study. The languages reported by bilinguals and bilingual musicians included English (*N* = 112, 100% of bilinguals and bilingual musicians), Spanish (*N* = 53, 47.3%), Korean (*N* = 15, 13.4%), Mandarin (*N* = 14, 12.5%), Chinese (unspecified) (*N* = 8, 7.1%), Arabic, Cantonese, and Polish (each *N* = 3, 2.7%), French (*N* = 2, 1.8%), Bengali, Czech, German, Gujarati, Hebrew, Japanese, Lithuanian, Marathi, Russian, Tamil, and Urdu (each *N* = 1, 0.9%). Twenty (17.9%) bilinguals and bilingual musicians learned English as the first language, 61 (54.4%) learned English as the second language, and 31 (27.7%) learned English and another language simultaneously. Mean age of acquisition of the second language (or both languages in the case of simultaneous bilinguals) was 4.39 (range = 0–14) years.

Musicians and bilingual musicians listed a variety of instruments, including Piano (*N* = 65, 58.6%), Voice (*N* = 45, 40.1%), Guitar (*N* = 34, 30.6%), Violin (*N* = 26, 23.4%), Flute (*N* = 20, 18.0%), Drums/Percussion (*N* = 11, 9.9%), Clarinet (*N* = 10, 9.0%), Saxophone (*N* = 8, 7.2%), Bass (*N* = 7, 6.3%), Trumpet (*N* = 6, 5.4%), Viola (*N* = 4, 3.6%), Cello, Recorder, and Xylophone (each *N* = 3, 2.7%), Guzheng, Oboe, and Ukulele (each *N* = 2, 1.8%), and Banjo, Bassoon, Erhu, French Horn, Trombone, and Tuba (each *N* = 1, 0.9%). Mean age of acquisition of the first learned instrument was 8.41 (range = 0–18) years (one participant indicated voice training within the first year). On average, musicians and bilingual musicians played 2.32 (range = 1–5) instruments and had taken 11.26 (range = 0–31) years of lessons.

Table 1 provides each group's demographic information with respect to male-to-female gender ratio, age, nonverbal IQ (performance subtests of the* Wechsler Abbreviated Scale of Intelligence*;* WASI*), and short-term memory (digit span subtest of the* Comprehensive Test of Phonological Processing*;* CTOPP*). (The following background data are unavailable for a subset of participants: gender (*N* = 1), nonverbal IQ (*N* = 5), and digit span (*N* = 3).) Measures of IQ and short-term memory were included in order to determine whether groups differed on other variables that are known to correlate with interference suppression [38, 39]. The* WASI* nonverbal IQ was derived from the block design and matrix reasoning subtests. In the block design subtest, participants quickly rearranged a set of blocks to copy a pattern. In the matrix reasoning subtest, participants saw a pattern with a missing element and chose the response option that best completed the pattern. The short-term memory score was derived from the* CTOPP* digit span task. In the digit span task, participants heard a series of numbers and had to repeat those numbers in the same order in which they heard them.

A chi-square analysis indicated no group differences in* male-to-female gender ratio*, *χ* ^2^(3, *N* = 218) = 2.81, *p* > 0.05. A one-way ANOVA with* age* as the dependent measure revealed an effect of Group, *F*(3,215) = 4.95, *p* < 0.05, with the bilingual musicians being younger than the bilinguals and controls (*p*s < 0.05, Bonferroni corrected). An effect of Group was also found in one-way ANOVAs on* short-term memory* performance (*F*(3,212) = 3.80, *p* < 0.05) and* nonverbal IQ* (*F*(3,210) = 2.65, *p* = 0.05), with participants in the bilingual group having lower digit spans than the other three groups (*p*s < 0.05, Bonferroni corrected) and marginally lower IQs than musicians (*p* = 0.067, Bonferroni corrected). Because the groups differed in age, digit span, and IQ, additional analyses of the Simon data were conducted to control for differences in these measures (described in Section 2.3 and presented at the end of Section 3).

### 2.2. Materials

**Figure 2:**
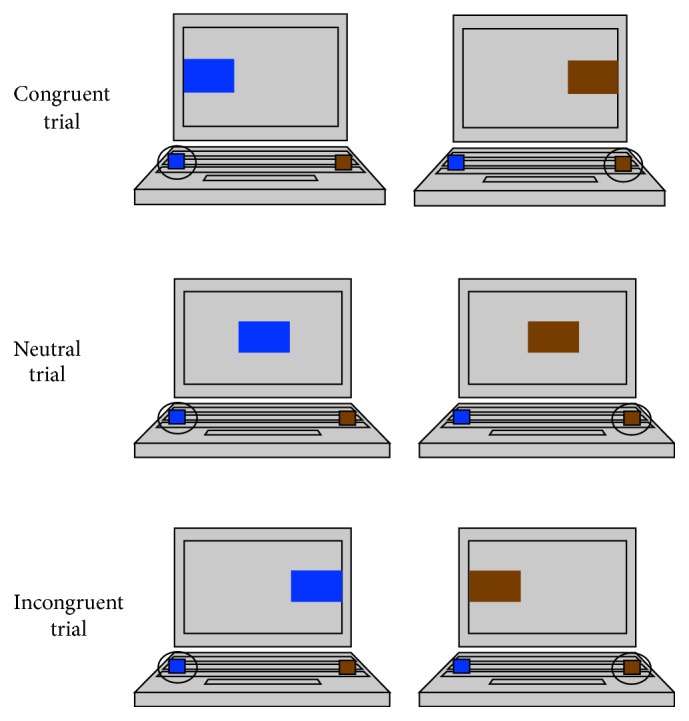
The three trial types (congruent, neutral, and incongruent) in the Simon task.

A visual-spatial Simon task [40] was used to measure interference suppression. The Simon task was chosen because it is well-validated, uses nonlinguistic and nonmusical stimuli, and has an effective control condition (i.e., when neutral trials are included as a baseline control condition, the task consistently elicits neutral response times that are slower than congruent response times and faster than incongruent response times). In the Simon task, a blue or brown rectangle was presented on the left, center, or right side of the computer screen. Participants were asked to push a button on the left side of the keyboard (the “A” key marked with a blue sticker) when the blue rectangle was presented and a button on the right side of the keyboard (the “L” key marked with a brown sticker) when the brown rectangle was presented, regardless of the spatial location of the rectangle. When the rectangle appeared on the same side as the response button (i.e., a blue rectangle on the left side of the screen or a brown rectangle on the right side of the screen), the trial was classified as congruent. When the rectangle appeared in the middle of the screen, the trial was classified as neutral. When the rectangle appeared on the side opposite to the response button (i.e., a blue rectangle on the right side of the screen or a brown rectangle on the left side of the screen), the trial was classified as incongruent.  Figure 2 provides a visual depiction of congruent, neutral, and incongruent trials. Participants completed 126 experimental trials (42 congruent trials, 42 neutral trials, and 42 incongruent trials) in a random order that was fixed across participants. In each trial, a fixation cross appeared for 350 ms, followed by a blank screen that was displayed for 150 ms, followed by a colored rectangle that was presented for 1500 ms, followed by a 850 ms blank screen serving as the intertrial interval. When an error was committed, an “X” was displayed on the screen for 1500 ms. When a correct response was made, no feedback was provided and the next trial began immediately. Prior to completing the 126 experimental trials, participants completed 24 practice trials, 8 of each trial type.

### 2.3. Data Analysis

Simon trials that were responded to incorrectly, that were not responded to within the 1500 ms response window, or that had a response time greater than 2.5 standard deviations from a participant's mean were removed from the dataset. This procedure led to the omission of 6% of all trials.

After removing these trials, response time data were submitted to a 4 × 3 mixed-design ANOVA, with Group (bilinguals, musicians, bilingual musicians, and controls) as the between-subjects independent variable and Congruency (congruent, neutral, and incongruent) as the within-subjects independent variable. (In a preliminary analysis, a 2 (language status: a bilingual, not a bilingual) × 2 (music status: a musician, not a musician) × 3 Congruency (congruent, neutral, and incongruent) ANOVA was conducted; this analysis yielded a three-way interaction, *F*(2,430) = 3.92, *p* < 0.05, providing a statistical justification for dividing participants into our 4 groups (bilinguals, musicians, bilingual musicians, and controls).) In the event of an interaction between Group and Congruency, follow-up ANOVAs were conducted on the interference effect (as well as the facilitation effect and Simon effect). The interference effect is calculated by subtracting response time on neutral trials from response time on incongruent trials. The facilitation effect is calculated by subtracting response time on congruent trials from response time on neutral trials. Note that the facilitation effect is difficult to interpret, as it may reflect a better ability to utilize the irrelevant but informative stimulus location on congruent trials [16, 23] or a worse ability to inhibit the irrelevant but informative stimulus location on congruent trials [41, 42]. The Simon effect is calculated by subtracting response time on congruent trials from response time on incongruent trials. The Simon effect is also difficult to interpret because it conflates facilitation and interference effects [41]. Nevertheless, Simon effect and facilitation effect data are presented to enable readers to compare our results to the results of other studies. Follow-up pairwise comparisons on the interference, facilitation, and Simon effects were conducted with Bonferroni corrections.

Five additional analyses were then conducted to verify the results of the primary analysis. First, ANCOVAs with IQ, digit span, and age as covariates were conducted due to group differences in these measures. Second, an analysis was conducted on a subset of the participants who were matched on IQ, digit span, and age. In this analysis, subsets were selected by randomly sampling 42 participants per group (*n* = 42 was chosen because the smallest group contained 42 participants) until the four groups did not differ significantly on IQ (*F* = 2.62, *p* > 0.05), digit span (*F* = 2.48, *p* > 0.05), or age (*F* = 2.30, *p* > 0.05). This random sampling procedure was conducted by first assigning a number to each participant in a group and then using a random number generator to select 42 participants from each of those groups. We repeated this procedure until one of the samples yielded *p* values above 0.05 for all three ANOVA comparisons (IQ, digit span, and age). In a third analysis, we entered neutral response times as a covariate (as a proxy of processing speed) in ANCOVAs, because processing speed may affect the magnitude of interference and facilitation effects and because there were nonsignificant trends of group differences in raw response times. In a fourth analysis, we conducted separate multiple linear regressions for the interference effect, the facilitation effect, and the Simon effect, with music proficiency, bilingual proficiency, and an interactive term of music and bilingual proficiency as the predictor variables; these analyses treat music and bilingual proficiency as continuous variables and provide a more fine-grained analysis than categorical analyses that collapse across individual differences in proficiency. In the fifth analysis, ANOVAs were conducted on accuracy on the Simon task in order to rule out a speed-accuracy trade-off.

## 3. Results

A Group (bilinguals, musicians, bilingual musicians, and controls) by Congruency (congruent, neutral, and incongruent) ANOVA conducted on response time data yielded a significant main effect of Congruency, *F*(2,430) = 453.58, *p* < 0.05, *η*_*p*2_ = 0.68, a marginally significant main effect of Group, *F*(3,430) = 2.50, *p* = 0.06, *η*_*p*2_ = 0.03, and a significant interaction between Group and Congruency, *F*(6,430) = 3.87, *p* < 0.05, *η*_*p*2_ = 0.05. (Table 2 presents the raw RTs for each group at each level of Congruency.) The* main effect of Congruency* demonstrated the validity of the Simon task and reflected that neutral trials were faster than incongruent trials (i.e., there was an interference effect), that congruent trials were faster than neutral trials (i.e., there was a facilitation effect), and that congruent trials were faster than incongruent trials (i.e., there was a Simon effect) (all *p*s < 0.05, Bonferroni corrected). The marginally significant* main effect of Group* indicated that groups might differ in overall response time, but Bonferroni corrected pairwise comparisons indicated no significant or marginally significant differences (all *p*s > 0.1). The significant* interaction between Group and Congruency* suggested differences between groups in the interference effect, facilitation effect, and/or Simon effect.

**Figure 3:**
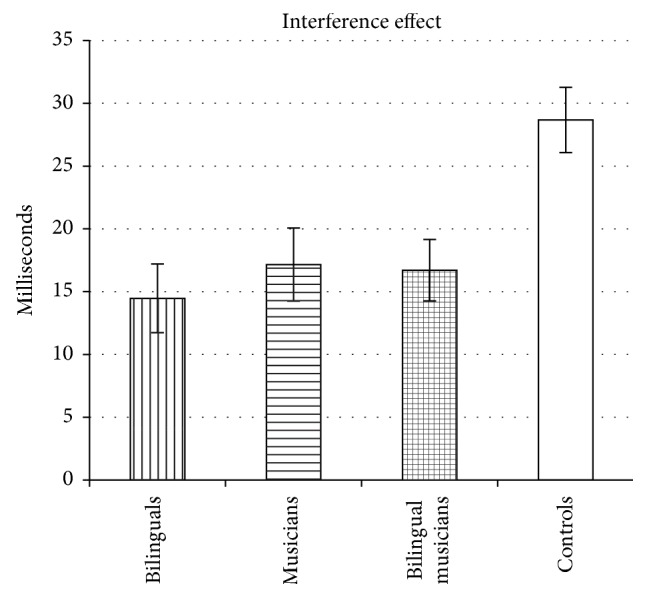
The mean interference effect (incongruent trials minus neutral trials) for bilinguals, musicians, bilingual musicians, and controls. Error bars represent the standard error of the mean.

**Figure 4:**
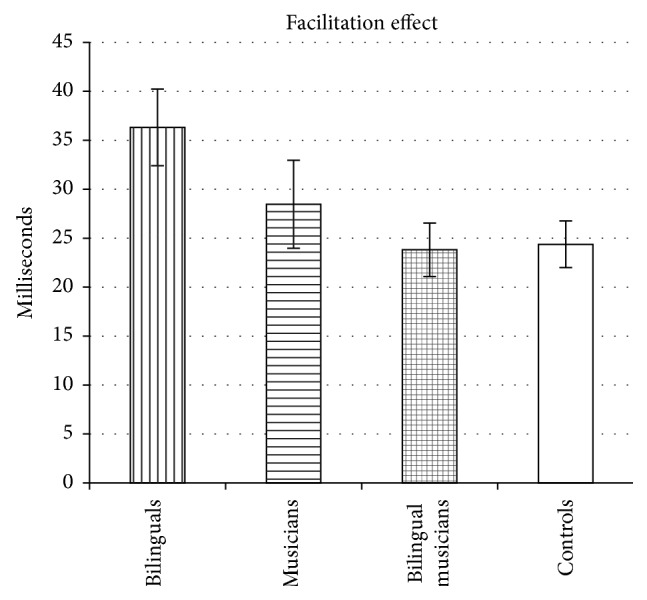
The mean facilitation effect (neutral trials minus congruent trials) for bilinguals, musicians, bilingual musicians, and controls. Error bars represent the standard error of the mean.

**Figure 5:**
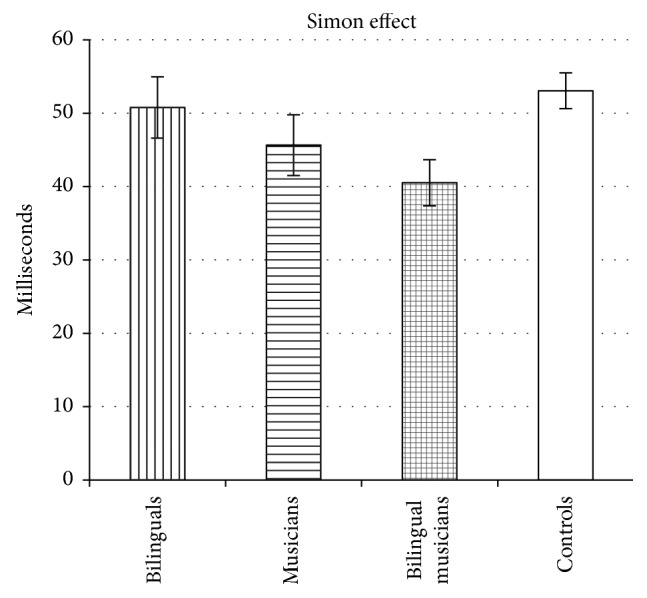
The mean Simon effect (incongruent trials minus congruent trials) for bilinguals, musicians, bilingual musicians, and controls. Error bars represent the standard error of the mean.

To follow up on the interaction between Group and Congruency, one-way ANOVAs were conducted on the interference effect as well as the facilitation effect and Simon effect. An ANOVA performed on the* interference effect* yielded a significant difference among groups, *F*(3,215) = 6.21, *p* < 0.05, *η*_*p*2_ = 0.08, with pairwise comparisons indicating that bilinguals, musicians, and bilingual musicians had smaller interference effects than controls, indicative of better interference suppression (all *p*s < 0.05, Bonferroni corrected). The interference effects for all 4 groups are displayed in Figure 3. An ANOVA performed on the* facilitation effect* yielded a significant difference among groups, *F*(3,215) = 2.93, *p* < 0.05, *η*_*p*2_ = 0.04, with pairwise comparisons indicating that bilinguals had a significantly larger effect than bilingual musicians (*p* < 0.05, Bonferroni corrected) and a marginally significantly larger effect than controls (*p* = 0.067, Bonferroni corrected). The facilitation effects for all 4 groups are displayed in Figure 4. An ANOVA performed on the* Simon effect* also yielded a significant difference among groups, *F*(3,215) = 3.23, *p* < 0.05, *η*_*p*2_ = 0.04. Pairwise comparisons revealed a smaller Simon effect in bilingual musicians relative to controls (*p* < 0.05, Bonferroni corrected). The Simon effects for all 4 groups are displayed in Figure 5.

Five additional analyses were then conducted to confirm the above results (see Section 2.3 for detailed explanations of how and why these analyses were conducted). First, we conducted ANCOVAs with IQ, digit span, and age as covariates in order to control for group differences in these measures; these analyses yielded a significant group difference in the* interference effect*, *F*(3,207) = 5.91, *p* < 0.05, with bilinguals, musicians, and bilingual musicians producing smaller interference effects than controls, reflecting enhanced interference suppression (all *p*s < 0.05, Bonferroni corrected). There was also a significant group difference in the* facilitation effect*, *F*(3,207) = 3.03, *p* < 0.05, with bilinguals producing larger facilitation effects than bilingual musicians (*p* < 0.05, Bonferroni corrected) (and marginally larger effects than controls, *p* = 0.097, Bonferroni corrected), as well as a significant group difference in the* Simon effect*, with bilingual musicians producing smaller Simon effects than controls (*p* < 0.05, Bonferroni corrected). Next, an analysis was conducted on a subset of the participants who were matched on IQ, digit span, and age. Analyses carried out on this subset yielded similar results (i.e., significant group differences in the* interference effect*, *F*(3,164) = 8.80, *p* < 0.05, with bilinguals, musicians, and bilingual musicians producing smaller (i.e., better) interference effects than controls, all *p*s < 0.05, Bonferroni corrected; significant group differences in the* facilitation effect*, *F*(3,164) = 2.81, *p* < 0.05, with bilinguals producing larger facilitation effects than bilingual musicians, *p* < 0.05, Bonferroni corrected; and significant group differences in the* Simon effect*, *F*(3,164) = 4.10, *p* < 0.05, with bilingual musicians producing smaller Simon effects than controls, *p* < 0.05, Bonferroni corrected). In the next analysis, neutral response time was entered as covariate (as a proxy of processing speed) in an ANCOVA in order to control for the potential effects of processing speed on the interference effect and facilitation effect. This ANCOVA replicated the* interference effect* results (i.e., significant group differences, *F*(3,214) = 6.41, *p* < 0.05, with bilinguals, musicians, and bilingual musicians producing smaller (better) interference effects than controls, all *p*s < 0.05, Bonferroni corrected). (Another way to control for processing speed is to calculate proportional interference effects (interference effect divided by response time on neutral trials). Analyses of proportional interference effects yielded the same results (i.e., significant group differences, *F*(3,215) = 6.63, *p* < 0.001, with bilinguals, musicians, and bilingual musicians producing smaller interference effects than controls).) However, when taking processing speed into consideration, there were no significant or marginally significant differences in the* facilitation effect*, *F*(3,214) = 1.83, *p* > 0.1.

Next, we conducted linear multiple regression analyses of the interference effect, facilitation effect, and Simon effect, with bilingual proficiency, music proficiency, and a bilingual proficiency/music proficiency interaction term as predictor variables. When the* interference effect* was entered as the dependent variable, bilingual proficiency was a significant predictor (beta weight = −0.32, *p* < 0.05) and music proficiency was a marginally significant predictor (beta weight = −0.21, *p* = 0.059), while the interactive term was not significant (beta weight = 0.03, *p* > 0.1). These results confirm that higher bilingual proficiency and higher music proficiency were associated with smaller (i.e., better) interference effects (but that bilingual and music proficiency do not have additive effects). When the* facilitation effect* was entered as the dependent variable, bilingual proficiency was a significant predictor (beta weight = −0.34, *p* < 0.05) and the interactive term was a marginally significant predictor (beta weight = −0.04, *p* = 0.057), while music proficiency was not significant (beta weight = 0.03, *p* > 0.1). When the* Simon effect* was entered as the dependent variable, none of the predictor variables reached significance (*p*s > 0.1).

Lastly, analyses were conducted on the accuracy data. Accuracy was high overall (mean congruent accuracy = 98.23%, mean neutral accuracy = 97.71%, and mean incongruent accuracy = 93.23%). An ANOVA yielded a significant main effect of Congruency, *F*(2,430) = 100.60, *p* < 0.001, *η*_*p*2_ = 0.32, reflecting that incongruent trials were responded to less accurately than congruent and neutral trials (*p*s < 0.05, Bonferroni corrected), but no main effect of Group, *F*(3,430) = 1.63, *p* > 0.1, *η*_*p*2_ = 0.02, or interaction between Group and Congruency, *F*(6,430) = 1.03, *p* > 0.1, *η*_*p*2_ = 0.01. The lack of a difference between groups in accuracy suggests that group differences in interference effect response times were not due to a speed-accuracy trade-off.

## 4. Discussion

In the current study, we examined the effects of bilingual and musical experience on executive control using a nonlinguistic, nonmusical, visual-spatial Simon task. Our results revealed lower interference effects in bilinguals, musicians, and bilingual musicians (relative to controls), indicative of enhanced interference suppression. These results were observed across all analyses and lend support to the idea that experience can drive plasticity in cognitive functions.

In addition to the interference effect, we reported participants' Simon effect score, which is often used to assess interference suppression. The Simon effect may not provide a pure measure of interference suppression (because it is influenced by congruent facilitation); consequently, this measure may conceal true bilingual and musician advantages in interference suppression. Indeed, the Simon effect results masked the enhancement in bilinguals and musicians because of variability in the facilitation effect across groups.

Aside from our primary finding of better interference suppression in bilinguals, musicians, and bilingual musicians, three secondary results are worth noting. First, in some analyses, bilinguals produced larger facilitation effects than both the controls and bilingual musicians. Larger facilitation effects in bilinguals relative to controls have previously been reported in the literature [16]. However, in the current study, facilitation differences did not hold up after factoring out the effects of processing speed, age, IQ, and short-term memory. Thus, in our study, the facilitation results may have been due to cognitive and demographic differences among the groups. Another result of interest is that bilingual musicians had smaller Simon effects than controls, while bilinguals and musicians did not differ from controls. This result may suggest that the combination of bilingual and music experience (relative to bilingual or music experience alone) is necessary to develop advantages on some aspects of the Simon task. However, Simon effects are difficult to interpret because they conflate the ability to utilize congruent cues (i.e., facilitation effects) with the ability to ignore incongruent cues (i.e., interference effects) [41] and thus further research in needed to clarify this finding. A third result relates to overall response speed on the Simon task. Although not significant, there was a numerical trend toward bilinguals responding more slowly than other groups (particularly, musicians and bilingual musicians). This slight, but not reliable, delay in bilinguals' response times contrasts with some previous work reporting significantly faster overall response times for bilinguals (e.g., [1]) but is consistent with several other studies (e.g., [23, 43, 44]). This trend appears to be due in part to the bilingual sample's lower IQ and short-term memory scores; indeed, the differences in overall response time are considerably smaller when controlling for IQ and short-term memory. Importantly, the primary finding of a bilingual enhancement in interference suppression does not seem to be due to differences in response time, as this enhancement is still observed after controlling for response time in our analyses.

Bilinguals' enhanced interference suppression ability observed in the present study may be due to their need to reduce competition from cross-linguistic activation of the nontarget language during comprehension and/or production. In support of this explanation, Blumenfeld and Marian [37] reported a correlation between the degree of activation of cross-linguistic similar-sounding words during speech comprehension (e.g., activation of the Spanish word* cartera* when hearing the English word “carpet”) and the level of enhanced interference suppression in bilinguals.

Musicians also demonstrated enhanced interference suppression ability in the current study. However, the reasons why musicians show better interference suppression ability are less clear than in bilinguals. As noted earlier, one possibility is that the musician advantage derives in part from a similar mechanism as the bilingual advantage. That is, analogous to how bilinguals activate similar-sounding words in the nontarget language during language comprehension, musicians may activate similar-sounding nontarget melodies during music comprehension [24, 25]. This melody activation is not specific to musicians in the same way that word activation is not specific to bilinguals. However, just as bilinguals have a larger number of words to suppress, musicians have a larger number of melodies to suppress (and likely higher frequency of certain melodies). The additional melodies that musicians activate could make the task of melody identification more difficult and may therefore serve as a good exercise for suppression mechanisms. Consistent with the notion that melody identification is more difficult for musicians, musicians were shown to identify melodies at a later point in time than nonmusicians [24]. This challenging practice of inhibiting interference from nontarget melodies during music comprehension may improve musicians' cognitive control abilities, similar to how management of nontarget words may enhance bilinguals' cognitive control abilities.

While there may be similarities in the mechanisms behind bilingual and musician advantages, notable differences may also exist. For example, during speech production, bilinguals need to select a single language at a time because both of their languages use the same output modality (the mouth) and thus cannot be produced simultaneously (e.g., the word,* cat*, and the Spanish word for cat,* gato*, cannot be produced concurrently). The need to limit speech production to one language at a time may train executive control abilities and contribute to a bilingual advantage [2]. When this restriction of one output modality is removed, as in speech-sign bimodal bilinguals who use two different output modalities (the mouth and the hands), the benefits to executive control are reduced [45]. (Note, however, that bimodal bilinguals still face inhibitory demands during comprehension like unimodal bilinguals, which may result in some executive control benefits; [46, 47].) Like bimodal bilinguals, musicians are able to produce in their language and instrument at the same time; even voice musicians can simultaneously incorporate both melody and words in their productions. While musicians may not have the same constraints on production as unimodal bilinguals, they nevertheless develop similar enhancements in interference suppression. This finding suggests that the source of enhanced interference suppression in musicians may differ in some ways from that of unimodal bilinguals.

One potential source of musicians' enhancement comes from the OPERA (Overlap, Precision, Emotion, Repetition, and Attention) hypothesis of musical training [48]. According to this hypothesis, musical training involves focused attention, for example, in selectively attending to the fine acoustic details of sound sequences. Increased selective attention can contribute to better interference suppression, as reducing interference from irrelevant cues can be accomplished through selective attention to the relevant cue and/or inhibition of the irrelevant cue. It is possible then that improved interference suppression in musicians may arise from the need to attend to fine acoustic details while limiting interference from factors that may impede attention to the details of sound.

Similar to bilinguals and musicians, bilingual musicians also demonstrated better interference suppression ability than controls. However, bilingual musicians did not outperform bilinguals or musicians, suggesting that the benefits of bilingualism and musicality may not be additive. A possible reason why bilingual musicians did not show further gains over bilinguals or musicians is that bilingualism and musicianship alone may reach the upper limits of interference suppression in many younger adults, precluding any further benefit of experience with both.

One limitation of the current study is that strong claims about a causal relationship between linguistic/musical experience and executive control cannot be made, given that participants were not randomly assigned to groups. Random assignment to life-long bilingual, musical, or bilingual-musical experience is not feasible. Nevertheless, due to a lack of random assignment, it is possible that the bilinguals, musicians, and bilingual musicians in the current study were cognitively advantaged before their extensive training in a second language and/or a musical instrument. However, considering our cognitive measures, this possibility seems unlikely for both musicians and bilinguals: musicians did not outperform controls on measures of IQ and short-term memory, and bilinguals were actually disadvantaged relative to controls in short-term memory.

Another limitation relates to potential group differences in socioeconomic status (SES). Because SES was not directly measured in the current study, it is possible that the groups differed in SES and that this difference contributed to the results (see [4] for a discussion). While SES was not directly measured, IQ test performance was measured, and IQ test performance has been shown to correlate highly with SES [49]. Group differences in IQ were controlled for in the present study through both matched-groups analyses and analyses of covariance.

A third limitation of the current study is the way in which bilingual and musical proficiency were measured. Because we tested participants with a diverse set of linguistic and musical backgrounds, it was not feasible to collect objective proficiency measures for each language and instrument. Instead we used subjective measures, which have been shown to correlate significantly with objective measures [33, 50]. Nevertheless, objective measures are more precise and should be used in future studies to confirm the current findings.

Future studies should also address some of the questions that arise from the current finding of enhanced interference suppression in bilinguals, musicians, and bilingual musicians. One such question is the extent to which bilingual and musician advantages derive from similar mechanisms. Another is what may be some of the reasons why bilingual musicians do not have interference suppression abilities that are above those of bilinguals and musicians.

In closing, the current study took a behavioral approach to cognitive plasticity by assessing whether adults with second language and/or musical experience have advantages in executive control. The results indicate enhanced interference suppression in bilinguals, musicians, and bilingual musicians relative to monolingual nonmusicians. We conclude that learning a second language or playing a musical instrument has benefits that extend beyond the specific domains of language and music to more general nonlinguistic cognitive function, including core skills like executive control. Because executive control abilities are related to a broad range of competencies [51, 52], these findings have implications for education practices by encouraging support for second language and music instruction.

## Figures and Tables

**Table 1:** Participant demographics (means and standard deviations).

	Bilinguals	Musicians	Bilingual musicians	Controls
Gender	9 M, 34 F	10 M, 32 F	23 M, 46 F	15 M, 49 F
Age^*∗*^	22.30 (4.05)	22.21 (3.42)	20.60 (2.86)	22.88 (4.03)
IQ (WASI)^*∗*^	109.16 (8.59)	114.74 (9.92)	113.55 (9.56)	111.67 (11.39)
Digit span (CTOPP)^*∗*^	16.12 (3.00)	17.69 (1.54)	17.47 (2.76)	17.55 (2.46)

WASI = Wechsler Abbreviated Scale of Intelligence.

CTOPP = Comprehensive Test of Phonological Processing.

Asterisks indicate significant group differences.

**Table 2:** Response times across groups and conditions.

	Bilinguals	Musicians	Bilingual musicians	Controls
Congruent RT	444.79 (73.19)	416.25 (71.32)	418.69 (60.40)	434.36 (72.25)
Neutral RT	481.11 (77.35)	444.72 (80.70)	442.50 (69.00)	458.73 (80.55)
Incongruent RT	495.58 (75.66)	461.88 (80.78)	459.21 (68.64)	487.41 (80.54)
